# Reactive atrial‐based antitachycardia pacing therapy reduces atrial tachyarrhythmias

**DOI:** 10.1111/pace.13696

**Published:** 2019-04-29

**Authors:** George H. Crossley, Luigi Padeletti, Steven Zweibel, J. Harrison Hudnall, Yan Zhang, Giuseppe Boriani

**Affiliations:** ^1^ Vanderbilt Heart and Vascular Institute Nashville Tennessee; ^2^ Cardiology Department, I.R.C.C.S. MultiMedica Sesto San Giovanni Milano Italy; ^3^ Hartford Healthcare Heart and Vascular Institute Hartford Hospital Hartford Connecticut; ^4^ Medtronic plc Mounds View Minnesota; ^5^ Cardiology Division, Department of Biomedical, Metabolic and Neural Sciences University of Modena and Reggio Emilia, Modena University Hospital Modena Italy

**Keywords:** atrial fibrillation, atrial flutter, atrial tachycardia, defibrillator, pacemaker, pacing, reactive ATP, resynchronization

## Abstract

**Background:**

Reactive atrial‐based antitachycardia pacing (rATP) aims to terminate atrial tachyarrhythmia/atrial fibrillation (AT/AF) episodes when they spontaneously organize to atrial flutter or atrial tachycardia; however, its effectiveness in the real‐world has not been studied. We used a large device database (Medtronic CareLink, Medtronic, Minneapolis, MN, USA) to evaluate the effects of rATP at reducing AT/AF.

**Methods:**

Pacemaker, defibrillator, and resynchronization device transmission data were analyzed. Eligible patients had device detected AT/AF during a baseline period but were not in persistent AT/AF immediately preceding first transmission. Note that 1:1 individual matching between groups was conducted using age, sex, device type, pacing mode, AT/AF, and percent ventricular pacing at baseline. Risks of AT/AF events were compared between patients with rATP‐enabled versus control patients with rATP‐disabled or not available in the device. For matched patients, AT/AF event rates at 2 years were estimated by Kaplan‐Meier method, and hazard ratios (HRs) were calculated by Cox proportional hazard models.

**Results:**

Of 43,440 qualifying patients, 4,203 had rATP on. Matching resulted in 4,016 pairs, totaling 8,032 patients for analysis. The rATP group experienced significantly lower risks of AT/AF events lasting ≥1 day (HR 0.81), ≥7 days (HR 0.64), and ≥30 days (HR 0.56) compared to control (P < 0.0001 for all). In subgroup analysis, rATP was associated with reduced risks of AT/AF events across age, sex, device type, baseline AT/AF, and preventive atrial pacing.

**Conclusions:**

Among real‐world patients from a large device database, rATP therapy was significantly associated with a reduced risk of AT/AF. This association was independent of whether the patient had a pacemaker, defibrillator, or resynchronization device.

## 1 INTRODUCTION

Atrial fibrillation (AF) is the most prevalent heart rhythm disorder with approximately 5.1 million lives being affected in the Unites States.^1^ Prevalence varies by age, with an approximate 5% increase in risk per year above the age of 65^2^ and by sex, with 60% being male.^3^ Although many episodes are asymptomatic, AF is associated with poor quality of life and increased risks of heart failure, dementia, stroke, and death.^4^, ^5^, ^6^ Costs of AF to the United States healthcare system total $26 billion annually, and the incremental cost for a single patient is $8,705.^7^ As the disease progresses to persistent episodes and to a permanent condition, symptoms worsen, comorbidities become more prevalent, and risks of thromboembolism, acute heart failure decompensation, and death increase.^3^, ^5^

AF is irregular, typically originates from the pulmonary veins, and, as such, requires cardioversion to terminate persistent episodes. AF is not susceptible to pace‐termination; however, slower organized rhythms such as atrial flutter or atrial tachycardia can often be terminated by antitachycardia pacing (ATP).^8^ Atrial tachyarrhythmia (AT/AF) episodes can spontaneously transition between AF and atrial flutter/tachycardia, even in patients with chronic AF.^9^ Reactive atrial‐based ATP therapy (rATP, Medtronic, Minneapolis, MN, USA), found in cardiac implantable electronic devices, aims to terminate AT/AF episodes when they spontaneously organize to atrial flutter or to atrial tachycardia, thereby slowing the AT/AF disease progression from paroxysmal to persistent and permanent forms. The randomized MINERVA trial was the first study to test the effects of rATP, and reported that use of rATP in patients with pacemakers and a history of paroxysmal or persistent AT/AF were associated with a lower incidence of progression to persistent and permanent AF.^10^ In addition, use of rATP reduced early recurrence of AF and was associated with more frequent positive atrial remodeling (>10% reduction in left atrial diameter).^11^ AF‐related hospitalization, AF‐related emergency department visits, and cardioversions were also significantly reduced.^10^, ^12^ However, MINERVA's population was limited to pacemaker recipients without complete atrioventricular block. Thus, we set out to study the real‐world effectiveness of rATP at reducing AT/AF occurrence and subsequently preventing AT/AF progression in patients with pacemakers, implantable cardioverter defibrillators (ICDs), and cardiac resynchronization (CRT) devices using a large nationwide device database.

## 2 METHODS

### 2.1 Study design and database

The study was a retrospective cohort assessment designed to evaluate the effectiveness of rATP therapy at reducing occurrence of AT/AF and subsequently preventing progression of AT/AF in patients with pacemakers, ICDs, and CRT devices. Risks of paroxysmal, persistent, and longstanding‐persistent AT/AF events based on device detected daily AT/AF burden were compared between patients with rATP‐enabled versus patients with rATP‐disabled or not available in the device. Transmission data were extracted from the deidentified CareLink database on August 23, 2016. The database stores pacemaker, ICD, CRT‐defibrillator, and CRT‐pacemaker data transmitted via the Medtronic remote monitoring server and includes data on patient age, sex, implant date, device make and model, and data parameters (e.g., rATP, pacing mode, preventive atrial pacing), and diagnostics and episodes from the device. Diagnostic data used in this study were daily AT/AF burden and percentage ventricular pacing (VP). These data were continuous as the devices maintain up to 14 months of storage capacity. The device‐reported AT/AF burden is determined in a consistent manner across devices and is highly accurate, where appropriate detection of AT/AF episodes and duration sensitivity are >95%, regardless of right atrial lead position.^13^ Thus, adjudication of AT/AF episodes was deemed unnecessary. The CareLink database does not collect clinical parameters outside of the aforementioned items (e.g., medications, comorbidities, treatments, outcomes).

### 2.2 Patients

Patients were ≥18 years old and implanted with dual‐chamber pacemakers, dual‐chamber ICDs, CRT‐pacemakers, and CRT‐defibrillators. Devices implanted from January 1, 2012 or later were included, and only data from the patient's first device was used. The date of first CareLink transmission is called Day 0. We use “the first transmission” and “Day 0” interchangeably in this report. In an effort to align with the MINERVA design,^14^ eligible patients had a history of AT/AF at baseline but were not in persistent AT/AF immediately preceding Day 0. Specifically, patients had to have at least one day with 5 minutes or more of AT/AF during the baseline period of up to 1 year preceding Day 0 but were excluded if they had >23 hours of AT/AF consecutively from Day minus 7 through Day 0. Further, eligible patients had to have transmitted data beyond Day 0 for up to 2 years. Patients were identified as a rATP subject if they had the rATP feature turned on for all transmissions, or they were identified as the control subjects if they had rATP off for all transmissions or were implanted with devices not capable of delivering atrial‐ATP.

To control for imbalances between the two groups, we performed individual matching using baseline data that are known risk factors for AT/AF development. Patients were matched 1:1 for age, sex, device type, pacing mode, percentage VP, and AT/AF at baseline. Patients implanted with CRT devices were also matched on programming of the AdaptivCRT feature.

### 2.3 Mechanism of rATP

The rATP feature is a second‐generation algorithm designed to opportunistically terminate AT/AF rhythms when they spontaneously organize to atrial flutter or to an atrial tachycardia, even in persistent episodes. Upon detection of an AT/AF episode, the device monitors rhythm transitions based on regions of atrial cycle lengths and regularity. Up to three ATP pacing therapies (Ramp or Burst+) are delivered for each region that the episode may transition to. Cycle length regions are 50‐ms wide for regular rhythms and 100–150‐ms wide for irregular rhythms. Furthermore, if the AT/AF episode persists, a time interval feature allows for counters to reset; thus, ATP may be reapplied at programmable durations (e.g., every 7 hours). While we acknowledge that ATP cannot terminate true AF, the strength of the algorithm lies in its ability to redeliver ATP when it detects a change in the patient's rhythm to a moment when the rhythm might be more vulnerable to termination with ATP (e.g., atrial flutter). Performance of rATP, as reported by Padeletti et al., demonstrated a median efficacy of 44.4%, which was higher in episodes of long cycle length and regular rhythm and in episodes with many rhythm transitions.^15^

### 2.4 Endpoints

The aim of the study was to demonstrate that rATP was associated with a lower risk of AT/AF events, including paroxysmal, persistent, and longstanding‐persistent AT/AF. We used the same AT/AF endpoints from the randomized MINERVA trial.^10^ They were time to first AT/AF event lasting ≥1 day (paroxysmal), ≥7 consecutive days (persistent), and ≥30 consecutive days (longstanding‐persistent). All the AT/AF events were based on device‐detected daily AT/AF burden. In addition, days with >23 hours of AT/AF burden were counted and classified within every 30 days from Day minus 30 to up to 2 years of follow‐up, and the effects of rATP on this longitudinal discrete endpoint were evaluated. ATP efficacy was also determined in the rATP group using AT/AF episode data.

### 2.5 Statistical analysis

Individual matching was conducted through a Greedy algorithm. This process ensured that, for each rATP subject, the matched control had exactly the same values for categorical matching variables (sex, device type, pacing mode, AT/AF up to 1 year preceding Day 0, and AdaptivCRT) and was within the matching margins for continuous matching variables (age and percentage VP). Details of matching variables can be found in the Supplementary Material.

Primary analyses of study endpoints focused on the matched subjects. Time to an event started from Day 0 and ended at the date that the event occurred the first time for subjects who experienced such an event or the date of last documented daily AT/AF record during up to 2 years of follow‐up for those who did not experience such an event. For events lasting more than one day, the first day was used as the date of occurrence. Event rates at 2 years were estimated by Kaplan‐Meier method.

Effects of rATP on each time‐to‐event endpoint were evaluated using Cox proportional hazard models. Covariates considered in the models were group, age (<65 and ≥65 years), sex, device type, AT/AF during a baseline period of up to 1 year preceding Day 0, duration from implant to Day 0 (≤5 and >5 months), AT/AF burden from Day minus 30 to Day 0, and programming status of preventive atrial pacing therapies including atrial preference pacing (APP), atrial rate stabilization (ARS), and postmode switch overdrive pacing (PMOP) at Day 0. The last three sets of covariates were included to adjust for the differences in them between rATP and control subjects after the matching. Final models were determined using a backward selection. Hazard ratios (HRs) of rATP from subgroup analysis are presented in forest plots. Frailty models were conducted to account for the correlation in matched pairs and verify the results from Cox models.

To investigate the transitions in patients’ AT/AF burden level over time, the AT/AF burden within every 30 days starting from 30 days before to 720 days after Day 0 was classified into four categories (0, 1–6, 7–29, and 30 days of >23 hours of AT/AF burden). The data are illustrated by stacked bar charts showing the percentages of subjects with different AT/AF burden levels within every 30 days in the rATP and control groups. It was analyzed using the Generalized Estimating Equations (GEE) approach with multinomial link function and independent working correlation structure. Covariates considered in this GEE analysis were time, group, age, sex, device type, AT/AF up to 1 year preceding Day 0, duration from implant to Day 0, and APP, ARS, and PMOP at Day 0 .

ATP efficacy on AT/AF episodes was estimated in the rATP group using the GEE method with binomial link function. ATP efficacy was based on the success of the last AT/AF therapy delivered in an AT/AF episode.

SAS statistical software version 9.4 (SAS Institute, Cary, NC, USA) and R (an open source statistical software, https://www.r-project.org) were used. A P‐value of < 0.05 was considered statistically significant.

## 3 RESULTS

### 3.1 Patients

There were 43,440 patients from 3,439 centers who met eligibility criteria (Supplementary Figure S1). Most were from centers in the United States (42,765, 98.4%), and the rest were from centers in Australia (632, 1.5%) and New Zealand (33, 0.08%), and unknown (10, 0.02%). rATP was enabled in 4,203 (9.7%) of the patients.

Baseline characteristics that were considered in the individual matching are shown in Table 1. As expected, in the full cohort of 43,440 patients, rATP patients had more AT/AF at baseline. For example, 85.8% of rATP patients had at least one day with an hour or more of AT/AF versus 75.8% in the control group. Relative to control, a larger proportion of rATP patients were implanted with a pacemaker and had their device programmed to MVP mode. The 1:1 individual matching resulted in 4,016 pairs, bringing the total number of subjects to 8,032 for the primary analyses.

**Table 1:** Characteristics of the patients at baseline*

	Full cohort (N = 43,440)		Matched cohort (N = 8,032)	
Variable	Reactive ATP group (N = 4,203)	Control group (N = 39,237)	Reactive ATP group (N = 4,016)	Control group (N = 4,016)
Age – years	73.0 ± 11.0	71.8 ± 11.5	73.4 ± 10.5	73.4 ± 10.5
Male sex – no. (%)	2,476 (58.9%)	24,442 (62.3%)	2,370 (59.0%)	2,370 (59.0%)
Device type – no. (%)				
Pacemaker	2,852 (67.9%)	23,688 (60.4%)	2,775 (69.1%)	2,775 (69.1%)
ICD	578 (13.8%)	6,287 (16.0%)	531 (13.2%)	531 (13.2%)
CRT†	773 (18.4%)	9,262 (23.6%)	710 (17.7%)	710 (17.7%)
Pacing mode – no. (%)				
DDD/R	1,514 (36.0%)	18,087 (46.1%)	1,408 (35.1%)	1,408 (35.1%)
DDI/R	24 (0.6%)	427 (1.1%)	6 (0.2%)	6 (0.2%)
MVP/R	2,664 (63.4%)	20,714 (52.8%)	2,602 (64.8%)	2,602 (64.8%)
AAI/R	1 (0.02%)	9 (0.02%)	0 (0.0%)	0 (0.0%)
AT/AF up to one year preceding Day 0 – no. (%)‡				
At least 1 day with ≥5 minutes, <1 hour	596 (14.2%)	9,483 (24.2%)	570 (14.2%)	570 (14.2%)
At least 1 day with ≥1 hour, <1 day	2,114 (50.3%)	19,380 (49.4%)	2,079 (51.8%)	2,079 (51.8%)
At least 1 day, <7 consecutive days	778 (18.5%)	6,257 (16.0%)	747 (18.6%)	747 (18.6%)
At least 7 consecutive days, <30 consecutive days	360 (8.6%)	2,388 (6.1%)	318 (7.9%)	318 (7.9%)
At least 30 consecutive days	355 (8.5%)	1,729 (4.4%)	302 (7.5%)	302 (7.5%)
Ventricular pacing ‐ %§				
Pacemaker	26.3 ± 39.2	34.5 ± 42.2	25.6 ± 39.2	25.6 ± 39.2
ICD	16.4 ± 32.3	15.1 ± 30.6	13.1 ± 30.0	13.0 ± 30.1
CRT	94.6 ± 14.2	92.5 ± 15.8	96.4 ± 9.4	96.5 ± 9.4

^*^Data at first transmission (Day 0). Plus‐minus values are means ± standard deviation.

^†^With or without defibrillator.

^‡^From device data up to 12 months preceding Day 0.

§From device data up to 30 days preceding Day 0.

AT/AF = atrial tachyarrhythmia; ATP = antitachycardia pacing; CRT = cardiac resynchronization therapy; ICD = implantable cardioverter defibrillator.

The individual matching resulted in nearly identical characteristics of matching factors between the matched patient groups (Table 1). Specifically, in each group, patients were mostly male (59.0%) and on average 73 years of age; 69.1%, 13.2%, and 17.7% had pacemakers, ICDs, and CRTs, respectively; 64.8% had MVP/R pacing mode at the first transmission. Among CRT patients, 57.5% did not have the AdaptivCRT feature available, 10.3% had Adaptive BiV, 21.4% had Adaptive BiV and LV, and 10.8% had Non‐Adaptive BiV (not shown in Table 1). During the up to 1‐year baseline period that preceded the first transmission, 7.9% and 7.5% of patients had >23 hours of daily AT/AF burden lasting for at least 7 and 30 consecutive days, respectively. Differences after matching were observed in the AT/AF burden from Day minus 30 to Day 0 (87.8% rATP vs 85.1% control with 0 days of >23 hours of AT/AF burden), duration from implant to Day 0 (median: 5.9 and 4.3 months in rATP and control subjects, respectively), and preventive atrial pacing therapies at Day 0 (9.3%, 9.7%, and 15.9% in rATP subjects vs 2.7%, 2.0%, and 8.3% in control subjects with APP, ARS, and PMOP being programmed on, respectively).

### 3.2 Time to AT/AF events

The incidence of AT/AF events were significantly reduced in the rATP group compared to control after controlling for other covariates in the main effect Cox models (P < 0.0001 for all, Table 2). Specifically, patients with rATP were 19% less likely to have an AT/AF event lasting ≥1 day (HR: 0.81; 95% confidence interval [CI], 0.74–0.88), 36% less likely to have an AT/AF event lasting ≥7 days (HR: 0.64; 95% CI, 0.57–0.73), and 44% less likely to have an AT/AF events lasting ≥30 days (HR: 0.56; 95% CI, 0.48–0.68). The frailty model results were consistent with those from Cox proportional hazard models (P < 0.0001 for all, Table 2). In the final Cox models, rATP effect remained highly significantly (P < 0.0001 for all, data not shown), while ARS and PMOP were not significant and hence removed from the final models and APP was slightly significant only in the final model for AF/AF lasting ≥1 day (P = 0.0469, data not shown).

**Table 2:** Risks of AT/AF between matched patient groups (N = 8,032)

	Number of subjects with event (2‐year Kaplan‐Meier event rate)		Cox proportional hazard model*		Frailty model*	
Event	Reactive ATP group (N = 4,016)	Control group (N = 4,016)	Hazard ratio (95% CI)	P‐value	Hazard ratio (95% CI)	P‐value
AT/AF ≥1 day	1,123 (38.4%)	1,370 (43.0%)	0.81 (0.74–0.88)	<0.0001	0.79 (0.72–0.87)	<0.0001
AT/AF ≥7 days	537 (20.4%)	857 (28.9%)	0.64 (0.57–0.73)	<0.0001	0.62 (0.55–0.71)	<0.0001
AT/AF ≥30 days	306 (12.2%)	584 (20.1%)	0.56 (0.48–0.66)	<0.0001	0.54 (0.46–0.64)	<0.0001

^*^Model contains main effects only: group, age, sex, device type, AT/AF up to 1 year preceding Day 0, duration from implant to Day 0, AT/AF from Day minus 30 to Day 0, and other atrial therapies (APP, ARS, and PMOP) on Day 0. AT/AF = atrial tachyarrhythmia; ATP = antitachycardia pacing; CI = confidence interval.

**Figure 1:**
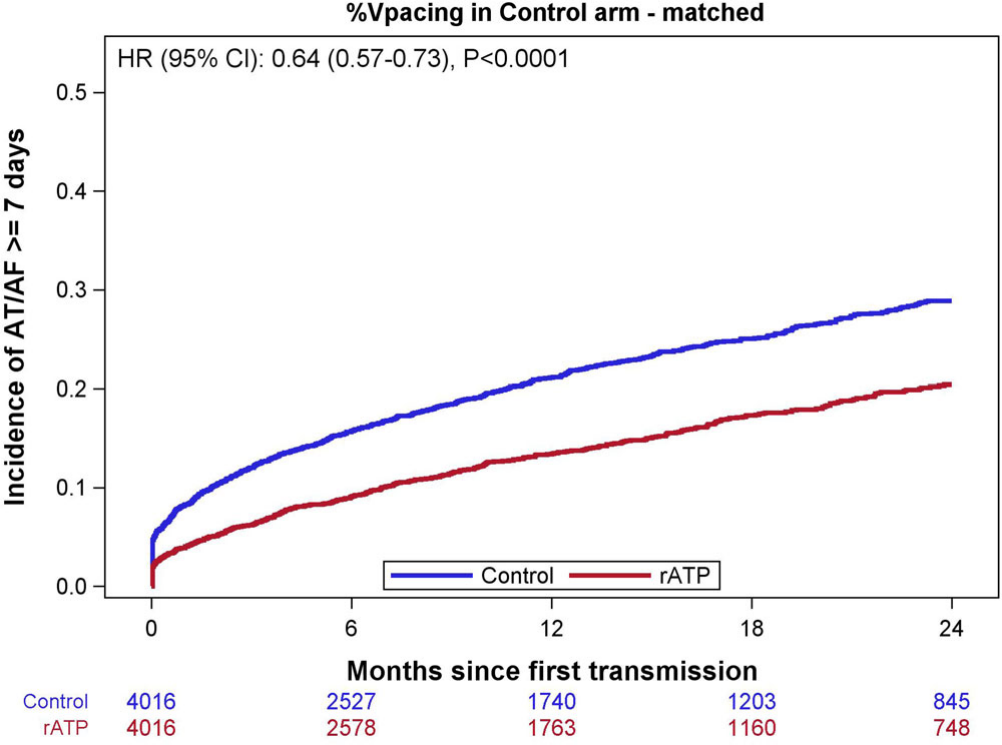
Kaplan‐Meier estimates of time to AT/AF events lasting ≥7 days for rATP and control groups. The hazard ratio of 0.64 (95% CI, 0.57–0.73) indicates a 36% decrease in the risk of having persistent AF among patients with rATP therapy on as compared to control. AT/AF = atrial tachyarrhythmia; CI = confidence interval; rATP = reactive atrial‐based antitachycardia pacing [Color figure can be viewed at http://wileyonlinelibrary.com]

Patients with rATP had an absolute reduction in the rates of AT/AF events lasting ≥1 day, ≥7 days, and ≥30 days at 2 years by 4.6%, 8.5%, and 7.9%, respectively. In Figure 1, the Kaplan‐Meier curves show a reduced risk of AT/AF ≥7 days in the rATP group immediately at Day 1, and the effects continued to progress out to 2 years.

**Figure 2:**
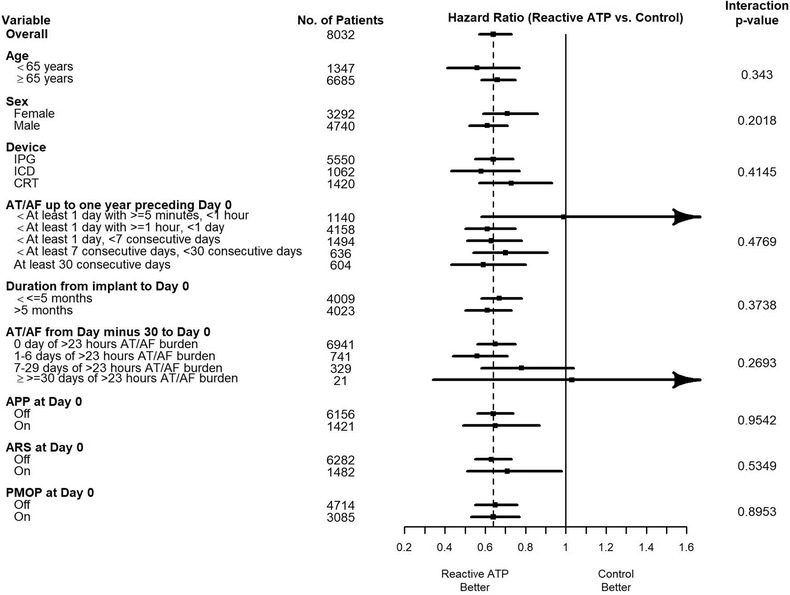
Forest plots comparing rATP to control in subgroups for time to AT/AF events lasting ≥7 days. Per Cox proportional hazard models. Vertical solid line corresponds to equal risk. Vertical dashed line is the overall hazard ratio. Horizontal solid lines are the 95% confidence intervals for the hazard ratios. AT/AF = atrial tachyarrhythmia; rATP = reactive atrial‐based antitachycardia pacing

Subgroup analyses were conducted to assess the effects of rATP on the risks of ≥1, ≥7, and ≥30 days of AT/AF across age, sex, device type, baseline AT/AF, and preventive atrial pacing at baseline. Figure 2 is the forest plot demonstrating that rATP was associated with reduced risk of ≥7 days of AT/AF for most subgroups. The 1‐day (Supplementary Figure S2) and 30‐day (Supplementary Figure S3) data are seen in the Supplementary Material. None of the interactions between group and individual factors was significant. Particularly, effects were consistent across pacemakers, ICDs, and CRT devices with the rATP group experiencing significantly reduced risks of having AT/AF events. For example, the risk of developing ≥7 days of AT/AF was significantly lower in rATP patients with pacemakers (HR: 0.64; 95% CI, 0.55–0.74), ICDs (HR: 0.58; 95% CI, 0.43–0.77), and CRT devices (HR: 0.73; 95% CI, 0.57–0.93). Significant rATP effects on the risk reduction of AT/AF events were also independent of APP, ARS, and PMOP being programmed on or not at the first transmission. Detailed results of the subgroups analysis for both the Cox proportional hazard and frailty models can be viewed in the Supplementary Material.

Cox regression analyses were also performed among the full cohort of 43,440 eligible patients, considering the same set of covariates as for the matched cohort. It was demonstrated in the main effect models that rATP was associated with significant reductions in AT/AF events lasting ≥1 day, ≥7 days, and ≥30 days (P < 0.0001 for all, Supplementary Table S4).

### 3.3 AT/AF burden within every 30 days over time

**Figure 3:**
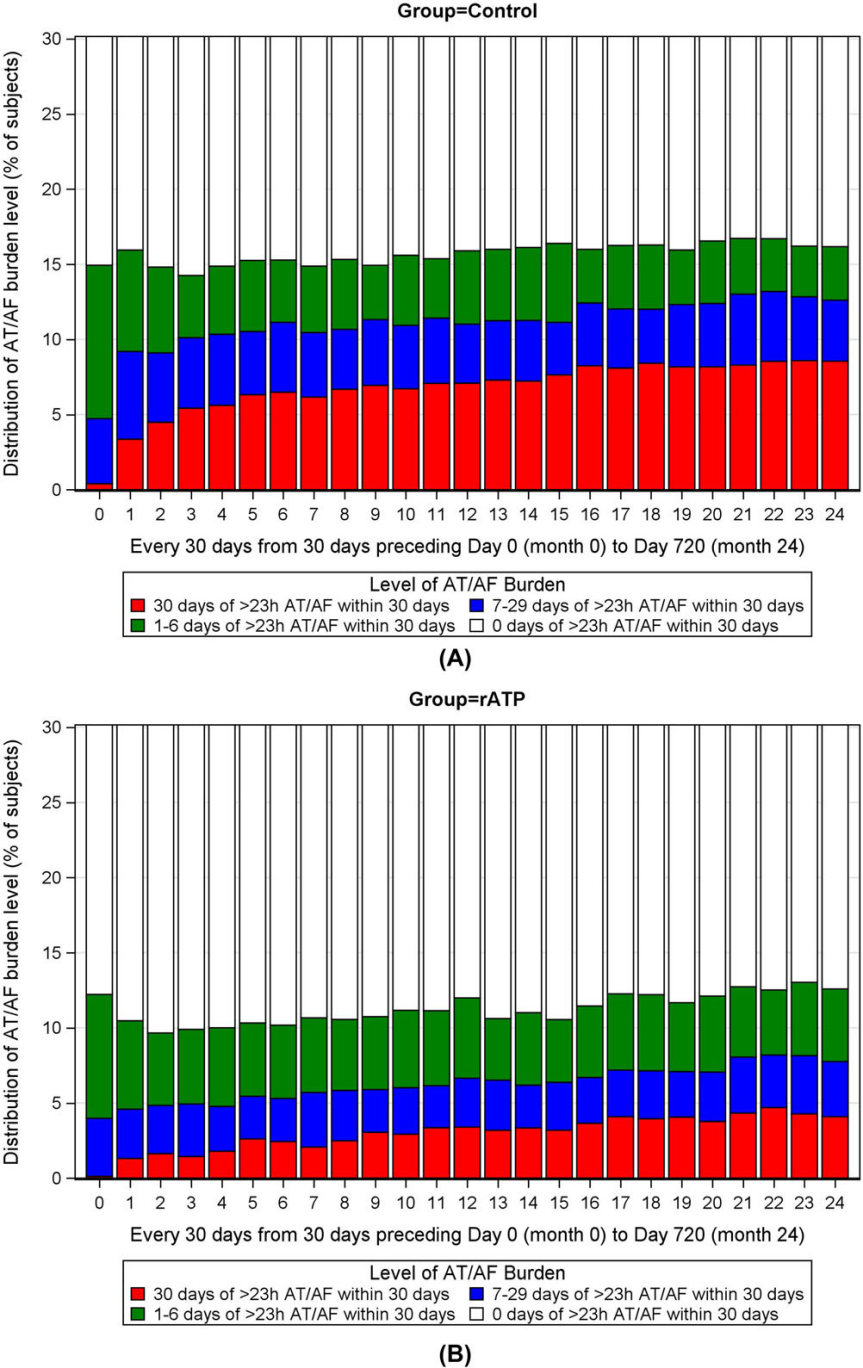
(A and B) Stacked bar charts for the distribution of AT/AF burden within every 30 days in rATP and control groups. Percentages of subjects with different AT/AF burdens sum up to 100% within every 30 days from 30 days before the first transmission (month 0) to 2 years after the first transmission (month 24). AT/AF = atrial tachyarrhythmia; rATP = reactive atrial‐based antitachycardia pacing [Color figure can be viewed at http://wileyonlinelibrary.com]

Stacked bar charts in Figure 3 show that the percentage of having zero days of > 23 hours of AT/AF in month 0 (i.e., within 30 days before the first transmission) was slightly higher in the rATP group (87.8%, 3,525/4,016) compared to the control group (3,416/4,016, 85.1%). For this reason, we controlled for the AT/AF burden from Day minus 30 to Day 0 in the Cox and frailty models for time‐to‐event endpoints. The bar charts also show the percentage of rATP patients having 30 days of > 23 hours of AT/AF within every 30 days from the first transmission to the end of 2 years of follow‐up was much lower than that of control patients. This difference was proven to be statistically significant in the GEE analysis. Specifically, when only considering time and group in the model, rATP patients were significantly less likely to have higher levels of AT/AF burden over time (risk ratio: 0.64, 95% CI: 0.57–0.73). After adjusting for all the covariates, the association of rATP with reduced risk of having higher levels of AT/AF burden was significant for all the categories of AT/AF burden up to 1 year preceding Day 0 (P < 0.05) except the lowest category (≥5 minutes but <1 hour, P = 0.9570).

### 3.4 ATP efficacy

In the rATP group, 198,838 out of 451,235 (44.1%) AT/AF episodes were terminated successfully by the last AT/AF therapy; the GEE estimated ATP efficacy on successfully terminating AT/AF episodes was 39.0% (95% CI: 38.1%–39.9%).

### 3.5 Case example

**Figure 4:**
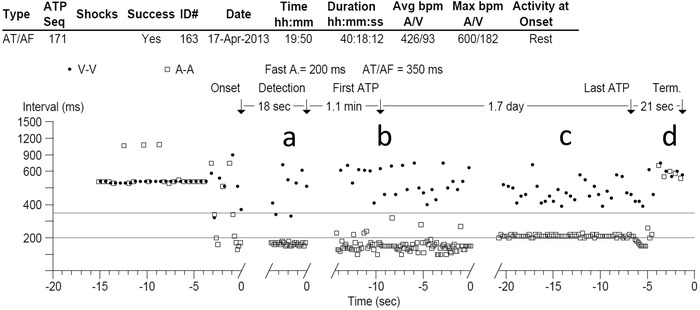
Patient case example of an AT/AF episode treated with rATP. The rhythm at onset is AF with a cycle length of 160–180 ms (A). The episode transitions to a very irregular rhythm with high variability in cycle length (B), during which first attempt at ATP has no effect. After 40 hours in episode duration, the rhythm shows a slowed and regular cycle length at 210 ms (C), and an attempt at ATP is successful at restoring sinus rhythm (D). AT/AF = atrial tachyarrhythmia; rATP = reactive atrial‐based antitachycardia pacing

A 48‐year‐old male was implanted with a Medtronic Protecta XT dual‐chamber ICD in February 2012; the device was programmed to MVP mode and rATP was enabled. In April 2013, the patient experienced an AT/AF episode (Figure 4). At onset, the rhythm was AF with a variable cycle length of 160–180 ms. First attempts at ATP failed while the rhythm was very irregular with higher variability in cycle length. After 40 hours and 18 minutes in episode duration, the cycle length slowed to 210 ms with the rhythm becoming more regular, and an attempt at ATP was successful at restoring sinus rhythm.

## 4 DISCUSSION

In this study, using prospectively acquired data from the deidentified Medtronic CareLink database, 43,440 patients with pacemakers, ICDs, and CRT devices that met eligibility criteria were identified, and rATP therapy was evaluated in an individually matched cohort of 4,016 rATP patients and 4,016 controls. rATP therapy was associated with significant reductions in the risk of having paroxysmal, persistent, and longstanding‐persistent AT/AF during 2 years of follow‐up. These reductions were consistent in subgroups, and they were independent of whether the patient had a pacemaker, ICD, or CRT device and whether preventive atrial pacing was activated at baseline. Our analysis also indicates reactive ATP is the principal driver of the observed AT/AF risk reductions. To our knowledge, these are the first real‐world data on the effectiveness of rATP therapy, and the first data of the therapy in patients with ICD and CRT devices. The data available had the characteristics of “big data,” that is, they were related to a large patient sample, although with limited granularity in terms of clinical profile.

Early studies of pacemaker/ICD integrated pacing therapies for AT/AF were promising. Prevention pacing, an atrial overdrive therapy aiming to suppress episode recurrence, was shown in several studies to reduce AT/AF.^16^, ^17^, ^18^ However, the positive findings from these trials have been overwhelmed by over a dozen studies showing no difference, the latest being the SAFE and ASSERT studies.^19^, ^20^ First‐generation atrial ATP therapy was found to be safe and effective at terminating episodes.^21^, ^22^ Yet, these effects failed to consistently demonstrate a reduction in AT/AF burden.

The latest trial to test pacing therapies for AT/AF, the randomized MINERVA study, was conducted in a selective pacemaker population and demonstrated superiority with prevention pacing plus second‐generation atrial ATP (rATP) versus standard pacemaker therapy at reducing the combined endpoint of death, cardiovascular hospitalization, and permanent AF. Results were driven by the effects on AF where the therapy reduced the risk of AT/AF >1 day by 34%, >7 days by 48%, and permanent AF by 61%.^10^ Subsequently, AF‐related hospitalizations, cardioversions, and emergency room visits were reduced, suggesting significant cost savings.^12^ These benefits were largely attributed to rATP therapy.^10^, ^15^ One unique aspect of the MINERVA trial was that rather than assessing the efficacy of rATP in terminating single episodes of AT/AF, more clinically valuable arrhythmic endpoints were evaluated, such as prevention of AF episodes of long duration. The novelty of rATP therapy includes the ability to redeliver ATP when the AT/AF rhythm changes cycle length and/or regularity and to retry ATP therapy over extended durations, with the aim to reduce the burden of AF and the occurrence of AF of longer duration. MINERVA showed that the algorithm was more effective when AT/AF episodes transitioned to slower and more regular rhythms.^15^ Furthermore, data on the reduction of early recurrence of AT/AF and reduction in left atrial diameter suggests reverse electrical and mechanical remodeling of the atria with use of rATP.^11^ These findings compelled us to test the effectiveness of the second‐generation ATP algorithm (rATP) in a large population and extend this evaluation to patients with ICDs and CRT devices.

Our findings not only corroborate the results from MINERVA, they suggest that the rATP effects may be equally beneficial regardless of the type of device. This has important implications due to the correlations of AF disease progression with outcomes, especially in patients with a CRT or ICD device who typically have a complex clinical profile that involves left ventricular dysfunction. Chiang et al.’s survey highlighted increased risks of comorbidities as AF progresses,^3^ and a recent meta‐analysis of 12 studies containing 99,996 patients reported that nonparoxysmal AF was associated with significant increases in thromboembolism and death.^23^ Recent findings from the ASSERT study reported a strong association between device‐detected subclinical AF progression and HF hospitalization.^24^ The CASTLE AF study also reported significant risk reductions in HF hospitalization and/or all‐cause death in the ablation arm along with a reduction in AT/AF burden.^25^ By taking into account all of the studies that examined the relationship between type and duration of device‐detected AF and risk of stroke, it appears that a dose–response association may exist between AF burden and the subsequent risk of stroke; therefore, reducing AT/AF burden may be indirectly considered as a potentially beneficial endpoint.^26^ Our study included patients with advanced AT/AF as 15% in the matched cohort had at least one persistent or longstanding‐persistent episode at baseline, yet rATP was effective immediately and effects persisted throughout the 2‐year follow‐up. This suggests that use of rATP does not need to be limited to patients with paroxysmal AF only.

### 4.1 Study limitations

The study has its limitations. This was a retrospective study, and similar to other analyses based on “big data,” there were uncollected clinical variables that we could not control for that include congestive heart failure, diabetes, hypertension, use of antiarrhythmic drugs, and other therapies for treating AT/AF. However, we controlled for key risk factors at baseline: age, sex, AT/AF, and percentage VP.^27^ Also, we controlled for device type and pacing mode which may relate to the patient's underlying arrhythmia condition and comorbidity (e.g., heart failure). There is potential for sampling bias as the data is taken from a single‐manufacturer's remote monitoring database largely from patients in the United States. Patients not followed by remote monitoring cannot be accounted for. Finally, patient outcomes such as cardiac function, permanent AF, stroke, and death could not be measured. For example, date of death was not available in the deidentified database either, hence its impact as a potential competing risk on AT/AF incidence could not be determined. Likewise, we did not have data on the use of antiarrhythmic drugs. The MINERVA trial has reported that baseline medications including the use of antiarrhythmic drugs were similar among the study arms,^10^ and patients in the DDDRP+MVP arm that had high reactive ATP efficacy experienced a significant reduction in developing permanent or persistent AF after controlling for antiarrhythmic drugs and other baseline medications.^15^ Although we cannot rule out all the potential bias with our data, the fact that the reactive ATP benefit that we observed in this observational study is aligned with the findings from the randomized MINERVA trial is reassuring.

### 5 CONCLUSION

In conclusion, among real‐world patients from a large database, rATP therapy was associated with a lower rate of AT/AF progression. Therapy effects were independent of whether the patient had a pacemaker, defibrillator, or CRT device.

## Supporting information

Supporting Information
