# Supplementary material for: Reactive atrial‐based antitachycardia pacing therapy reduces atrial tachyarrhythmias
Source: Pacing Clin Electrophysiol. 2019 Apr 29;42(7):970–9. doi: 10.1111/pace.13696 (PMC6850031; doi:10.1111/pace.13696)

**SUPPLEMENT**

**Baseline variables for individual matching:**

1. Age at Day 0: matching margin, ±2 years
2. Sex: female and male
3. Device type: IPG, ICD and CRT
4. Pacing mode at Day 0: AAI/R, DDI/R, DDD/R and MVP/R
5. Classification of AT/AF based on daily AT/AF burden during a baseline period of up to one year preceding Day 0:
   1. At least 1 day with ≥5 minutes AT/AF but <1 hour
   2. At least 1 day with ≥1 hour AT/AF but < 1 day
   3. At least 1 day AT/AF but <7 consecutive days
   4. At least 7 consecutive days AT/AF but <30 consecutive days
   5. At least 30 consecutive days AT/AF
6. Mean percentage VP within 30 days preceding Day 0: matching margin, ±5%
7. AdaptivCRT setup in CRT devices: no AdaptivCRT feature, NonAdaptiveBiV, AdaptiveBiV and AdaptiveBiVandLV.

**Supplementary Table S1: Sub-group Analysis: Time to AT/AF ≥1 Day between Matched Patient Groups**

| **Subgroup** |  | **Number of Subjects with Event**  **(2-year Kaplan-Meier Event Rate)** | | **Cox Proportional Hazard Model** | | **Frailty Model** | |
| --- | --- | --- | --- | --- | --- | --- | --- |
|  | **Number of Patients** | **Reactive ATP Group (N=4016)** | **Control Group (N=4016)** | **Hazard Ratio (95% CI)** | **Interaction**  ***P*-Value** | **Hazard Ratio (95% CI)** | **Interaction**  ***P*-Value** |
| **Overall** | 8032 | 1123 (38.4%) | 1370 (43.0%) | 0.81 (0.74-0.88) | - | 0.79 (0.72-0.87) | - |
| **Age** |  |  |  |  | 0.7766 |  | 0.7566 |
| <65 yrs | 1347 | 169 (34.0%) | 207 (38.0%) | 0.78 (0.63-0.97) |  | 0.77 (0.62-0.96) |  |
| ≥65 yrs | 6685 | 954 (39.4%) | 1163 (44.0%) | 0.81 (0.74-0.89) |  | 0.79 (0.72-0.88) |  |
| **Sex** |  |  |  |  | 0.8307 |  | 0.9346 |
| Female | 3292 | 418 (34.7%) | 501 (38.7%) | 0.80 (0.69-0.92) |  | 0.79 (0.69-0.92) |  |
| Male | 4740 | 705 (41.1%) | 869 (45.8%) | 0.81 (0.73-0.91) |  | 0.79 (0.70-0.89) |  |
| **Device** |  |  |  |  | 0.2269 |  | 0.1371 |
| Pacemaker | 5550 | 751 (36.7%) | 911 (40.9%) | 0.82 (0.73-0.91) |  | 0.80 (0.72-0.89) |  |
| ICD | 1062 | 142 (38.8%) | 194 (47.3%) | 0.68 (0.55-0.86) |  | 0.65 (0.52-0.82) |  |
| CRT | 1420 | 230 (45.1%) | 265 (47.8%) | 0.88 (0.72-1.08) |  | 0.88 (0.71-1.08) |  |
| **AT/AF up to one year preceding Day 0** |  |  |  |  | 0.2710 |  | 0. 3040 |
| At least 1 day with ≥5 minutes, <1 hour | 1140 | 45 (12.2%) | 47 (11.7%) | 1.05 (0.69-1.60) |  | 1.05 (0.69-1.61) |  |
| At least 1 day with ≥1 hour, <1 day | 4158 | 417 (30.4%) | 548 (35.5%) | 0.75 (0.66-0.86) |  | 0.74 (0.64-0.85) |  |
| At least 1 day, <7 consecutive days | 1494 | 392 (65.2%) | 450 (70.2%) | 0.88 (0.76-1.01) |  | 0.85 (0.73-0.99) |  |
| At least 7 consecutive days, <30 consecutive days | 636 | 160 (63.8%) | 174 (65.9%) | 0.83 (0.66-1.04) |  | 0.81 (0.64-1.03) |  |
| At least 30 consecutive days | 604 | 109 (52.8%) | 151 (64.0%) | 0.70 (0.54-0.91) |  | 0.70 (0.53-0.92) |  |
| **Duration from implant to Day 0** |  |  |  |  | 0.3108 |  | 0.1827 |
| ≤5 months | 4009 | 558 (40.6%) | 872 (47.2%) | 0.78 (0.69-0.87) |  | 0.75 (0.66-0.85) |  |
| >5 months | 4023 | 565 (36.9%) | 498 (37.6%) | 0.85 (0.74-0.96) |  | 0.84 (0.74-0.96) |  |
| **AT/AF from Day minus 30 to Day 0** |  |  |  |  | 0.9491 |  | 0. 8480 |
| 0 day of >23 hours AT/AF burden | 6941 | 760 (32.2%) | 892 (35.2%) | 0.82 (0.73-0.91) |  | 0.80 (0.72-0.90) |  |
| 1-6 days of >23 hours AT/AF burden | 741 | 244 (83.2%) | 323 (88.0%) | 0.79 (0.66-0.95) |  | 0.77 (0.63-0.94) |  |
| 7-29 days of >23 hours AT/AF burden | 329 | 115 (82.1%) | 140 (86.7%) | 0.76 (0.58-0.99) |  | 0.74 (0.55-0.99) |  |
| ≥30 days of >23 hours AT/AF burden | 21 | 4 (not estimable) | 15 (100.0%) | 0.92 (0.30-2.77) |  | 1.03 (0.29-3.66) |  |
| **Atrial Preference Pacing on Day 0** |  |  |  |  | 0.5727 |  | 0.7315 |
| Off | 6156 | 687(36.0%) | 1175 (41.9%) | 0.81 (0.74-0.90) |  | 0.80 (0.72-0.88) |  |
| On | 1421 | 375 (43.4%) | 107 (56.4%) | 0.76 (0.61-0.95) |  | 0.77 (0.60-0.97) |  |
| **Atrial Rate Stabilization on Day 0** |  |  |  |  | 0.8345 |  | 0.6658 |
| Off | 6282 | 709 (36.6%) | 1236 (43.0%) | 0.80 (0.73-0.89) |  | 0.79 (0.71-0.87) |  |
| On | 1482 | 389 (42.1%) | 79 (42.8%) | 0.83 (0.65-1.06) |  | 0.83 (0.63-1.08) |  |
| **Post Mode Switch Overdrive Pacing on Day 0** |  |  |  |  | 0.5396 |  | 0.6090 |
| Off | 4714 | 458 (35.0%) | 990 (42.1%) | 0.82 (0.74-0.92) |  | 0.80 (0.71-0.91) |  |
| On | 3085 | 640 (41.3%) | 335 (45.6%) | 0.78 (0.68-0.90) |  | 0.77 (0.66-0.90) |  |

**Supplementary Table S2: Sub-group Analysis: Time to AT/AF ≥7 Days between Matched Patient Groups**

| **Subgroup** |  | **Number of Subjects with Event**  **(2-year Kaplan-Meier Event Rate)** | | **Cox Proportional Hazard Model** | | **Frailty Model** | |
| --- | --- | --- | --- | --- | --- | --- | --- |
|  | **Number of Patients** | **Reactive ATP Group (N=4016)** | **Control Group (N=4016)** | **Hazard Ratio (95% CI)** | **Interaction**  ***P*-Value** | **Hazard Ratio (95% CI)** | **Interaction**  ***P*-Value** |
| **Overall** | 8032 | 537 (20.4%) | 857 (28.9%) | 0.64 (0.57-0.73) | - | 0.62 (0.55-0.71) | - |
| **Age** |  |  |  |  | 0.3430 |  | 0.3240 |
| <65 yrs | 1347 | 64 (14.2%) | 116 (22.7%) | 0.56 (0.41-0.77) |  | 0.54 (0.39-0.74) |  |
| ≥65 yrs | 6685 | 473 (21.8%) | 741 (30.2%) | 0.66 (0.58-0.75) |  | 0.64 (0.55-0.73) |  |
| **Sex** |  |  |  |  | 0.2018 |  | 0.0966 |
| Female | 3292 | 206 (18.9%) | 289 (24.0%) | 0.71 (0.59-0.86) |  | 0.70 (0.58-0.85) |  |
| Male | 4740 | 331 (21.5%) | 568 (32.3%) | 0.61 (0.52-0.71) |  | 0.58 (0.49-0.67) |  |
| **Device** |  |  |  |  | 0.4145 |  | 0.3379 |
| Pacemaker | 5550 | 316 (17.2%) | 517 (25.0%) | 0.64 (0.55-0.74) |  | 0.62 (0.53-0.72) |  |
| ICD | 1062 | 79 (24.3%) | 137 (35.2%) | 0.58 (0.43-0.77) |  | 0.53 (0.40-0.72) |  |
| CRT | 1420 | 142 (31.2%) | 203 (39.2%) | 0.73 (0.57-0.93) |  | 0.70 (0.55-0.91) |  |
| **AT/AF up to one year preceding Day 0** |  |  |  |  | 0.4769 |  | 0.4325 |
| At least 1 day with ≥5 minutes, <1 hour | 1140 | 28 (7.8%) | 31 (8.1%) | 0.99 (0.58-1.68) |  | 0.99 (0.58-1.68) |  |
| At least 1 day with ≥1 hour, <1 day | 4158 | 170 (14.1%) | 291 (20.0%) | 0.61 (0.50-0.75) |  | 0.60 (0.49-0.74) |  |
| At least 1 day, <7 consecutive days | 1494 | 163 (31.2%) | 262 (48.4%) | 0.63 (0.51-0.78) |  | 0.60 (0.49-0.75) |  |
| At least 7 consecutive days, <30 consecutive days | 636 | 104 (46.5%) | 146 (57.3%) | 0.70 (0.54-0.91) |  | 0.66 (0.50-0.87) |  |
| At least 30 consecutive days | 604 | 72 (34.6%) | 127 (55.2%) | 0.59 (0.43-0.80) |  | 0.56 (0.41-0.77) |  |
| **Duration from implant to Day 0** |  |  |  |  | 0.3738 |  | 0.5450 |
| ≤5 months | 4009 | 300 (23.8%) | 553 (32.4%) | 0.67 (0.58-0.78) |  | 0.64 (0.54-0.75) |  |
| >5 months | 4023 | 237 (17.8%) | 304 (24.5%) | 0.61 (0.50-0.73) |  | 0.60 (0.49-0.72) |  |
| **AT/AF from Day minus 30 to Day 0** |  |  |  |  | 0.2693 |  | 0.1902 |
| 0 day of >23 hours AT/AF burden | 6941 | 328 (15.9%) | 513 (21.7%) | 0.65 (0.56-0.75) |  | 0.64 (0.54-0.74) |  |
| 1-6 days of >23 hours AT/AF burden | 741 | 114 (46.2%) | 208 (68.7%) | 0.56 (0.44-0.71) |  | 0.51 (0.40-0.66) |  |
| 7-29 days of >23 hours AT/AF burden | 329 | 91 (67.3%) | 121 (78.2%) | 0.78 (0.58-1.04) |  | 0.73 (0.53-1.01) |  |
| ≥30 days of >23 hours AT/AF burden | 21 | 4 (not estimable) | 15 (100.0%) | 1.03 (0.34-3.12) |  | 1.10 (0.31-3.91) |  |
| **Atrial Preference Pacing on Day 0** |  |  |  |  | 0.9542 |  | 0.9419 |
| Off | 6156 | 337 (19.5%) | 728 (27.7%) | 0.64 (0.56-0.74) |  | 0.62 (0.54-0.72) |  |
| On | 1421 | 171 (22.4%) | 65 (39.6%) | 0.65 (0.49-0.87) |  | 0.62 (0.45-0.84) |  |
| **Atrial Rate Stabilization on Day 0** |  |  |  |  | 0.5349 |  | 0.7016 |
| Off | 6282 | 345 (19.9%) | 772 (28.8%) | 0.63 (0.55-0.73) |  | 0.62 (0.54-0.71) |  |
| On | 1482 | 182 (21.8%) | 49 (28.7%) | 0.71 (0.51-0.98) |  | 0.65 (0.46-0.92) |  |
| **Post Mode Switch Overdrive Pacing on Day 0** |  |  |  |  | 0.8953 |  | 0.8105 |
| Off | 4714 | 214 (18.3%) | 615 (27.9%) | 0.65 (0.55-0.76) |  | 0.63 (0.53-0.74) |  |
| On | 3085 | 313 (22.3%) | 214 (31.9%) | 0.64 (0.53-0.77) |  | 0.61 (0.50-0.75) |  |

**Supplementary Table S3: Sub-group Analysis: Time to AT/AF ≥30 Days between Matched Patient Groups**

| **Subgroup** |  | **Number of Subjects with Event**  **(2-year Kaplan-Meier Event Rate)** | | **Cox Proportional Hazard Model** | | **Frailty Model** | |
| --- | --- | --- | --- | --- | --- | --- | --- |
|  | **Number of Patients** | **Reactive ATP Group (N=4016)** | **Control Group (N=4016)** | **Hazard Ratio (95% CI)** | **Interaction**  ***P*-Value** | **Hazard Ratio (95% CI)** | **Interaction**  ***P*-Value** |
| **Overall** | 8032 | 306 (12.2%) | 584 (20.1%) | 0.56 (0.48-0.66) | - | 0.54 (0.46-0.64) | - |
| Age |  |  |  |  | 0.3102 |  | 0.3210 |
| <65 yrs | 1347 | 34 (7.7%) | 78 (15.5%) | 0.46 (0.30-0.70) |  | 0.45 (0.29-0.69) |  |
| ≥65 yrs | 6685 | 272 (13.2%) | 506 (21.1%) | 0.58 (0.49-0.68) |  | 0.56 (0.47-0.66) |  |
| **Sex** |  |  |  |  | 0.9202 |  | 0.9538 |
| Female | 3292 | 106 (10.1%) | 195 (16.6%) | 0.56 (0.43-0.71) |  | 0.55 (0.42-0.71) |  |
| Male | 4740 | 200 (13.7%) | 389 (22.5%) | 0.56 (0.47-0.68) |  | 0.54 (0.45-0.66) |  |
| **Device** |  |  |  |  | 0.3201 |  | 0.3027 |
| Pacemaker | 5550 | 168 (9.4%) | 342 (16.9%) | 0.53 (0.44-0.65) |  | 0.52 (0.43-0.64) |  |
| ICD | 1062 | 45 (15.3%) | 91 (23.8%) | 0.51 (0.35-0.74) |  | 0.48 (0.33-0.70) |  |
| CRT | 1420 | 93 (21.6%) | 151 (29.6%) | 0.68 (0.51-0.92) |  | 0.66 (0.49-0.89) |  |
| **AT/AF up to one year preceding Day 0** |  |  |  |  | 0.9226 |  | 0.8674 |
| At least 1 day with ≥5 minutes, <1 hour | 1140 | 15 (4.4%) | 21 (5.4%) | 0.76 (0.38-1.52) |  | 0.76 (0.38-1.51) |  |
| At least 1 day with ≥1 hour, <1 day | 4158 | 98 (8.1%) | 191 (13.2%) | 0.56 (0.43-0.72) |  | 0.55 (0.43-0.72) |  |
| At least 1 day, <7 consecutive days | 1494 | 86 (17.7%) | 167 (32.7%) | 0.56 (0.43-0.74) |  | 0.54 (0.41-0.72) |  |
| At least 7 consecutive days, <30 consecutive days | 636 | 62 (32.5%) | 108 (41.4%) | 0.55 (0.40-0.77) |  | 0.52 (0.37-0.73) |  |
| At least 30 consecutive days | 604 | 45 (21.1%) | 97 (45.0%) | 0.52 (0.35-0.77) |  | 0.50 (0.33-0.74) |  |
| **Duration from implant to Day 0** |  |  |  |  | 0.6925 |  | 0.5046 |
| ≤5 months | 4009 | 168 (14.8%) | 390 (23.1%) | 0.55 (0.45-0.67) |  | 0.52 (0.43-0.64) |  |
| >5 months | 4023 | 138 (10.1%) | 194 (16.3%) | 0.58 (0.46-0.74) |  | 0.58 (0.45-0.74) |  |
| **AT/AF from Day minus 30 to Day 0** |  |  |  |  | 0.8907 |  | 0. 7787 |
| 0 day of >23 hours AT/AF burden | 6941 | 185 (9.2%) | 341 (14.7%) | 0.57 (0.47-0.69) |  | 0.57 (0.46-0.69) |  |
| 1-6 days of >23 hours AT/AF burden | 741 | 64 (28.0%) | 132 (46.2%) | 0.51 (0.37-0.70) |  | 0.47 (0.34-0.66) |  |
| 7-29 days of >23 hours AT/AF burden | 329 | 55 (47.7%) | 97 (64.7%) | 0.60 (0.42-0.86) |  | 0.56 (0.38-0.82) |  |
| ≥30 days of >23 hours AT/AF burden | 21 | 2 (not estimable) | 14 (92.2%) | 0.62 (0.14-2.74) |  | 0.53 (0.10-2.66) |  |
| **Atrial Preference Pacing on Day 0** |  |  |  |  | 0.1901 |  | 0.1683 |
| Off | 6156 | 190 (11.6%) | 499 (19.5%) | 0.54 (0.45-0.64) |  | 0.52 (0.43-0.62) |  |
| On | 1421 | 97 (12.8%) | 36 (20.5%) | 0.71 (0.48-1.05) |  | 0.70 (0.46-1.05) |  |
| **Atrial Rate Stabilization on Day 0** |  |  |  |  | 0.4151 |  | 0.4637 |
| Off | 6282 | 196 (11.8%) | 526 (20.0%) | 0.55 (0.46-0.65) |  | 0.53 (0.44-0.63) |  |
| On | 1482 | 104 (13.0%) | 32 (19.2%) | 0.65 (0.44-0.98) |  | 0.62 (0.41-0.95) |  |
| **Post Mode Switch Overdrive Pacing on Day 0** |  |  |  |  | 0.2460 |  | 0.2728 |
| Off | 4714 | 117 (10.1%) | 430 (20.0%) | 0.52 (0.42-0.64) |  | 0.51 (0.41-0.63) |  |
| On | 3085 | 183 (14.0%) | 134 (20.0%) | 0.62 (0.49-0.79) |  | 0.60 (0.47-0.77) |  |

**Supplementary Table S4: Time to AT/AF among Unmatched Patients (N=43440)**

|  | **Number of Subjects with Event**  **(2-year Kaplan-Meier Event Rate)** | | **Cox Proportional Hazard Model^*^** | |
| --- | --- | --- | --- | --- |
| **Event** | **Reactive ATP Group (N=4203)** | **Control Group (N=39237)** | **Hazard Ratio (95% CI)** | ***P*-Value** |
| AT/AF ≥1 day | 1200 (38.9%) | 12774 (40.4%) | 0.79 (0.74-0.85) | <0.0001 |
| AT/AF ≥7 days | 589 (21.1%) | 8312 (28.0%) | 0.62 (0.56-0.68) | <0.0001 |
| AT/AF ≥30 days | 341 (12.8%) | 5823 (20.3%) | 0.53 (0.47-0.60) | <0.0001 |

*Model contains main effects only: group, age, sex, device type, AT/AF up to one year preceding Day 0, duration from implant to Day 0, AT/AF from Day minus 30 to Day 0, and other atrial therapies (APP, ARS and PMOP) on Day 0.

**Supplementary Figure S1:** Flow diagram of patients analyzed

Eligible Reactive ATP

patients (n=4203)

Eligible Control

patients (n=39237)

Not match to rATP patient (n=35221)

No matching Control patient (n=187)

1:1 matching via Greedy algorithm

Matched Reactive ATP patients (n=4016)

Matched Control patients (n=4016)

**Supplementary Figure S2:** Forest plot comparing rATP to Control in subgroups for Time to AT/AF events lasting ≥1 day


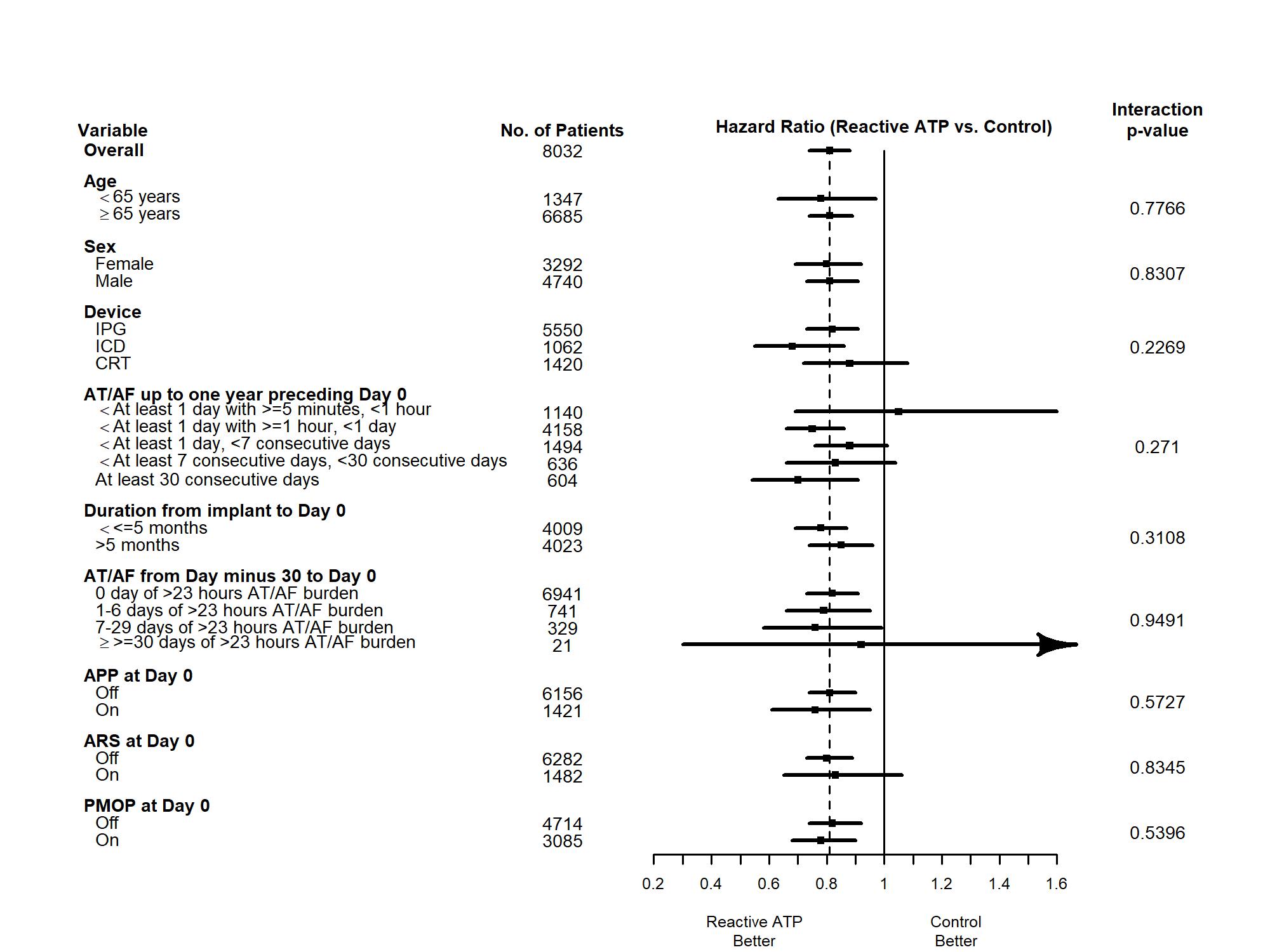


**Supplementary Figure S3:** Forest plot comparing rATP to Control in subgroups for Time to AT/AF events lasting ≥30 days


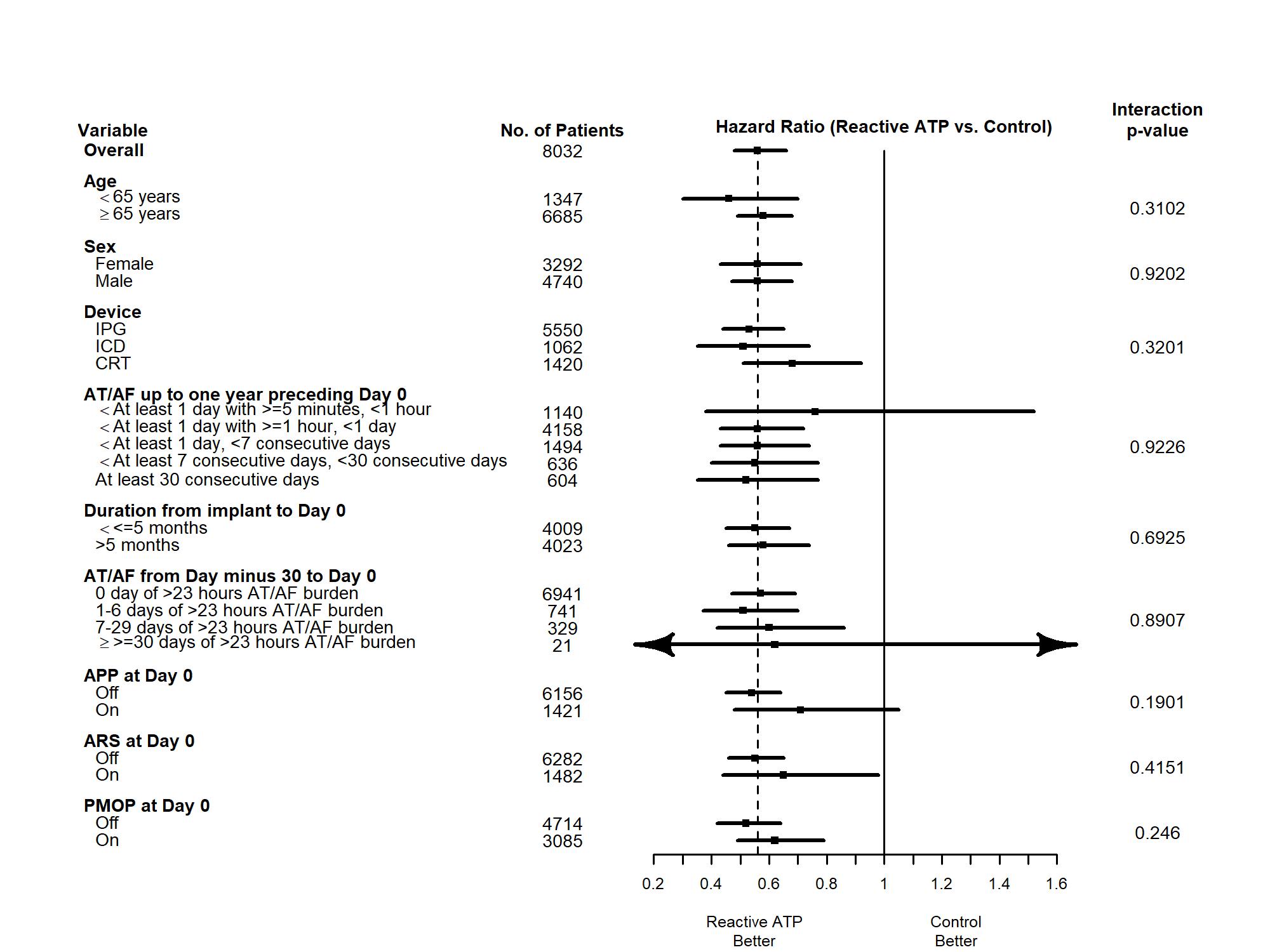

Supplement: Supplementary file 1 — Supporting Information [file PACE-42-970-s001.docx]
